# Effect of incorporation of walnut cake (*Juglans regia*) in concentrate mixture on degradation of dry matter, organic matter and production of microbial biomass *in vitro* in goat

**DOI:** 10.14202/vetworld.2015.1172-1176

**Published:** 2015-10-09

**Authors:** Mohsin Ahmad Mir, R. K. Sharma, Ankur Rastogi, Keshab Barman

**Affiliations:** 1 Division of Animal Nutrition, Shere-e-Kashmir University of Agricultural Sciences and Technology of Jammu (J&K), India; 2 Animal Nutrition Section, ICAR-NRC on Pig, Rani, Guwahati, Assam, India

**Keywords:** concentrate mixture, goat, *in vitro* dry matter digestibility, microbial biomass production, truly degradable organic matter, walnut cake

## Abstract

**Aim:**

This study was carried out to investigate the effect of incorporation of different level of walnut cake in concentrate mixture on *in vitro* dry matter degradation in order to determine its level of supplementation in ruminant ration.

**Materials and Methods:**

Walnut cake was used @ 0, 10, 15, 20, 25 and 30% level to formulate an iso-nitrogenous concentrate mixtures and designated as T_1_, T_2_, T_3_, T_4_, T_5_ and T_6_ respectively. The different formulae of concentrate mixtures were used for *in vitro* gas production studies using goat rumen liquor with wheat straw in 40:60 ratio. Proximate composition, fiber fractionation and calcium and phosphrous content of walnut cake were estimated.

**Result:**

The per cent IVDMD value of T1 and T2 diets was 68.42 ± 1.20 and 67.25 ± 1.37 respectively which was found highest (P<0.05) T3, T4, T5 and T6. Similar trend was also found for TDOM and MBP. Inclusion of walnut cake at 10% level in the concentrate mixture does not affect *in vitro* dry matter digestibility (IVDMD), truly degradable organic matter (TDOM, mg/200 mg DM), total gas production, microbial biomass production (MBP) and efficiency of microbial biomass production (EMP).

**Conclusion:**

It is concluded that walnut cake incorporation up to 10% level in the iso -nitrogenous concentrate mixture has no any negative effect on *in vitro* digestibility of dry matter (DM), TDOM, MBP, EMP and total gas production in goat.

## Introduction

Chronic feed deficits represent a major constraint to animal production in many developing countries. The situation manifests itself in poor animal performance, low growth rates, reduced reproductive efficiency, high mortality rates, etc. The genetic potential of many farm animals is inadequately exploited and the output of animal production, such as meat, milk, eggs, fibre and skins, often fall far short of national requirements. A way out of this situation has been found in identifying, characterizing and promoting utilization of unconventional feedstuffs. Main advantage of feeding unconventional feedstuffs to livestock is to have less dependency on conventional feedstuffs, thereby sparing them for alternate use and reduction in cost of feeding [1,2]. One of such promising unconventional feed ingredient is walnut cake.

Jammu and Kashmir contributes around 98% of the country’s output and annually producing about 86,263 tonnes from an area of 61,723 hectares.

The worldwide production of walnuts has been increasing rapidly in recent years, with the largest increase coming from Asia. The world produced a total of 2.55 million metric tonnes of walnuts in 2010. At present, China is the world’s largest producer of walnuts, with a total harvest of 1.06 million metric tonnes [3]. The other major producers of walnuts were (in the order of decreasing harvest): Iran (450,000 tonnes), United States (425,820 tonnes), Turkey (194,298 tonnes), Ukraine (96,900 tonnes), Mexico (110,605 tonnes), Romania (30,546 tonnes), India (40,000 tonnes), France (36,425 tonnes) and Chile (38,000 tonnes). The average worldwide walnut yield was about 3 metric tonnes per hectare, in 2010.

There are very few reports of the utilization of walnut cake/meal in livestock ration and it still remains an almost unstudied byproduct from the perspective of animal nutrition [4]. Utilization of walnut cake ruminant ration is not standardized. Hence, the present study has been carried out to find the level of incorporation of wall nut cake in ruminant ration using graded level in the ration *in vitro* using goat rumen liquor.

## Materials and Methods

### Ethical approval

This research was carried out after approval of Institutional Animal Ethics Committee of Shere-e-Kashmir University of Agricultural Sciences and Technology of Jammu.

### Source and processing of walnut cake

Freshly expelled walnut cake was collected in gunny bags and brought to the laboratory. The cake was sundried, ground and stored for analysis. Proximate composition, fiber fractionation and calcium and phosphrous content of walnut cake were estimated. Iso-nitrogenous concentrate mixtures (Table-1) containing graded levels of walnut cake (0 - 30% replacement) were formulated and subjected to *in vitro* gas production studies [5] using goat rumen liquor with concentrate: wheat straw at 40:60 ratio.

**Table-1:** Ingredient composition (%) of concentrate mixtures (w/w, as such basis).

Ingredients	T_1_	T_2_	T_3_	T_4_	T_5_	T_6_
Maize	30	25	22	20.5	18	15
Wheat bran	30	26	25	21.5	19.5	18
Mustard cake	37	36	35	35	34.5	34
Walnut cake	-	10	15	20	25	30
Mineral mixture	2	2	2	2	2	2
Salt	1	1	1	1	1	1

T_1_, T_2_, T_3_, T_4_, T_5_ and T_6_ represent 0, 10, 15, 20, 25 and 30% incorporation of walnut cake in the concentrate mixtures respectively

### Proximate analysis and fiber fractionation

Proximate analysis of walnut cake sample was done as per AOAC [6] and fiber fractions [Neutral detergent fiber (NDF) and Acid detergent fiber (ADF)] were done as per standard method [7].

On the day of incubation, the mixture of rumen liquor and particulate matter (approximately 60:40) was collected from local slaughter house into pre-warmed CO_2_ filled thermos and carried to the laboratory. The rumen fluid was bubbled with CO_2_ gas for few minutes and then mixed in a laboratory blender at medium speed to remove microbes attached to particulate matter. Rumen liquor was then strained through a double layer of muslin cloth. Strained liquor was then added to the buffer media (contains disodium hydrogen carbonate-9.8 g, sodium hydrogen phosphate – 9.3 g, sodium chloride-0.47 g, potassium chloride-0.57 g, magnesium chloride-0.06 g per 1000 ml distilled water with pH range of 6.7 to 6.8) when the media became colourless. Handling of rumen liquor was done under continuous flushing with CO_2_.

### Filling of syringes and incubation

The buffered rumen fluid (30 ml) was dispensed to each syringe containing 200 mg sample by a marked self made dispenser. After recording initial volume (± 0.5 ml), the syringes were placed in the incubator maintained at 39°C. The syringes were shaken by hand intermittently at 3 hours interval. All incubations were run in triplicate and four syringes with buffered rumen fluid were incubated as blanks. A zero hour blank in duplicate was also kept during dispensing of buffered rumen fluid into syringes. Standard was also run in triplicate with wheat straw alone as incubated sample. At the end of incubation (24 h) the amount of gas produced was measured by reading the position of the plug and the contents of the syringes were analyzed further.

### Determination of substrate degradation and microbial bio-mass production

The contents of the syringes were transferred to 500 ml spoutless beakers by repeated washings with neutral detergent solution without sodium sulphite [7]. The contents were then refluxed for 1 h to extract the microbial matter from the undegraded feed [8] and the residue was recovered in pre-weighed sintered crucibles. After drying the crucibles (with residue) at 100°C to constant weight, ashing was done at 400°C to 500°C for 2 h. *In vitro* dry matter degradability (IVDMD), Truly degradable organic matter (TDOM), microbial biomass production (MBP), efficiency of microbial biomass production (EMP) and Partition factor (PF) were calculated as follows:

TDOM = Feed (OM) incubated - residue (OM)
MBP = TDOM - (2.2 x net gas volume)
EMP = {TDOM - (2.2 x net gas volume)} x 100/TDOM
PF = TDOM/net gas volume

### Statistical analysis

Generalised linear model analysis of variance procedure was used for *in vitro* trial results and the means having significant difference were ranked as per Duncan’s multiple range test [9].

## Results and Discussion

### Proximate composition and fiber fractions of walnut cake

The percent OM and CP were found to be 93.30 and 15.17, respectively. The NDF and ADF content of the walnut cake was 41.33% and 28.07%, respectively. The CP content of concentrate mixture ranged from 17.57 in T1 to 18.14 in T6 and other fall within this range of variation (Table-2).

**Table-2:** Proximate composition (mean±SE)* and fiber fractionation of concentrate mixture and walnut cake.

Attribute	T_1_	T_2_	T_3_	T_4_	T_5_	T_6_	Walnut cake
Moisture	10.08±0.22	9.91±0.25	9.82±0.27	9.74±0.28	9.65±0.29	9.57±0.30	8.56±0.19
OM	90.06±0.23	90.11±0.18	90.13±0.16	90.16±0.13	90.18±0.11	90.20±0.09	93.30±0.10
CP	17.58±0.06	17.69±0.09	17.75±0.10	17.90±0.11	18.01±0.12	18.14±0.14	15.17±0.53
EE	4.02±0.22	4.86±0.19	5.28±0.18	5.69±0.17	6.11±0.15	6.53±0.14	12.15±0.87
TA	6.94±0.23	6.89±0.18	6.87±0.16	6.84±0.13	6.82±0.10	6.80±0.08	6.73±0.07
AIA	2.47±0.05	2.70±0.08	2.83±0.10	2.92±0.12	3.04±0.13	3.17±0.15	2.93±0.07
NDF	9.72±0.62	12.88±0.50	14.54±0.43	15.98±0.37	17.56±0.31	19.20±0.25	41.33±1.33
ADF	23.47±0.08	23.64±0.08	24.11±0.08	23.59±0.08	23.68±0.08	23.97±0.08	28.07±0.67
Ca	-	-	-	-	-	-	4.70±0.26
P	-	-	-	-	-	-	0.66±0.01

* All values are means of sample analysis in triplicate and are on DM basis except moisture. OM=Organic matter, CP=Crude protein, EE=Ether extract, TA=Total ash, AIA=Acid insoluble ash, NDF=Neutral detergent fiber, ADF=Acid detergent fiber, Ca=Calcium, P=Phosphorus, -=Not estimated, T_1_, T_2_, T_3_, T_4_, T_5_ and T_6_ represent 0, 10, 15, 20, 25 and 30% incorporation of walnut cake in the concentrate mixtures respectively

The moisture level of walnut cake recorded in the present study is well below 10-11% level, making it safe for long term storage. Unlike other oil cakes routinely used for livestock feeding, the moderate CP content (15.17%) and high crude fat content (12.15%) of walnut cake make it similar in composition to grain byproducts like wheat or rice bran. However, the fibre content of walnut cake (41.33% NDF and 28.07% ADF) is much higher than most of the conventional concentrate supplements including brans. Similarly olive cake also contained high fiber content [10]. Further, unlike other concnetrate supplements, it is rich in calcium and poor in phosphorus leading to a metabolically distastrous calcium: Phosphorus ration of about 7:1.

The results of the present study are in contrast to most of the available mentions of chemical composition of walnut cake in referred literature [11-13]. The locally available walnut cake used in the present study is low in protein and phosphorus as compared to the previously reported composition and seems to be high in lignocellulose content although none of the previous studies have reported fibre fractions and the comparisons were made taking reported crude fibre content in consideration.

It has been reported that the main nutrients of walnut oil meal - protein, fat and fibre - are extremely variable: The nutritive value of walnut oil meal ranges between that of the high-energy walnut kernels (50-60% oil) and the high fibre, low-energy shells. The nutritional value of walnut oil meal depends on the extraction process. The shells may or may not be removed prior to extraction, the kernels may or may not be toasted, there may be one or two mechanical pressing steps, cold or hot [4].

A French two-step process has been described as yielding two types of oil meals: The first pressing gives a relatively high fat (20%) and medium protein (32%) oil cake, whitish and containing kernel fragments, while the second pressing yields a darker (yellow-brown) product, containing less kernel particles, less oil (10-12%) and more protein (37%) [14] collected in France tend to support the notion that French walnut oil meals contain generally high levels of protein, low levels of fibre (5-10) (due to prior dehulling) and highly variable levels of oil (6-45%).

American processes seem to yield products containing much less protein (13-17%) and fat (6-10%) and a high amount of fibre (crude fibre 27-33%) [15-17]. It is reported by McGregor [15] that walnut meal contain twice the amount of fibre and half the protein, a composition barely more nutritious than that of walnut shells and it is this composition that was observed in the present study.

### *In vitro* degradation

The composite ration comprising of different iso-nitrogenous concentrate mixtures containing variable levels of walnut cake (L_10_, L_15_, L_20_, L_25_ and L_30_ containing 10%, 15%, 20%, 25% and 30% of walnut cake, respectively) on a wheat straw based diet regimen was tested against control (L_0_; conventional concentrate mixture) for *in vitro* dry matter degradation.

The per cent IVDMD of composite diet without the inclusion of walnut cake (control) was found to be highest at 68.42, which was comparable to that of L_10_ (67.25) and was significantly higher (P<0.01) from IVDMD at other inclusion levels, which were similar to each other (Table-3). The trend was same with respect to TDOM (mg/200 mg DM) and MBP (mg/200 mg DM), although, no effect of walnut cake inclusion was observed on gas production (ml/200 mg DM), EMP (% TDOM) and PF.

**Table-3:** *In vitro* dry matter degradation (mean±SE) of composite rations comprising of different concentrate mixtures containing variable levels of walnut cake with wheat straw in 40:60 ratio.

Rations*	IVDMD (%)	TDOM (mg/200 mg DM)	Gas production (ml/200 mgDM)	MBP (mg/200 mg DM)	EMP (% TDOM)	PF
L_0_{(Maize 30%, WC 0%, MOC 37%, WB 30%) 40:WS 60}	68.42^a^±1.20	132.13^a^±2.90	18.83±2.22	90.69^a^±5.50	68.61±3.72	7.60±1.03
L_10_{(Maize25%, WC10%, MOC 36%, WB 26) 40:WS 60}	67.25^a^±1.37	130.29^a^±4.17	19.5±1.55	87.39^a^±4.62	67.02±2.45	6.87±0.53
L_15_{(Maize 22%, WC15%, MOC 35%, WB 25%) 40:WS 60}	60.92^b^±2.26	113.96^b^±4.09	18.67±1.16	72.89^b^±6.24	63.43±3.28	6.30±0.63
L_20_{(Maize 20.5%, WC 20%, MOC 35%, WB 21.5%) 40:WS 60}	60.17^b^±1.21	117.68^b^±1.33	20.61±0.95	72.34^b^±2.92	61.40±2.04	5.78±0.30
L_25_{(Maize 18%, WC 25%, MOC 34.5%, WB 19.5%) 40:WS 60}	60.25^b^±1.43	116.30^b^±3.06	19.33±2.35	73.76^b^±3.93	63.68±3.72	6.34±0.57
L_30_ {(Maize 15%, WC 30%, MOC 34%, WB 18%) 40:WS 60}	60.58^b^±1.01	113.27^b^±3.97	18.67±2.00	72.21^b^±4.13	63.88±3.17	6.31±0.51
p value	0.000	0.010	0.970	0.018	0.618	0.454

ab Means bearing different superscripts within a column differ significantly (p≤0.01).

* WC=Walnut cake, MOC=Mustard oil cake, WB=Wheat bran, WS=Wheat straw, IVDMD=*In vitro* dry matter degradability, TDOM=Truly degradable organic matter, MBP=Microbial biomass production, EMP=Efficiency of microbial biomass production, PF=Partition factor

The composite ration comprising of different iso-nitrogenous concentrate mixtures containing variable levels of walnut cake on a wheat straw based diet regimen when tested against control via *in vitro* gas production study revealed that walnut cake is having a depressing effect over *in vitro* utilization of composite ration (Table-3), which becomes significant at 15% or higher inclusion levels of walnut cake in concentrate mixture. The depressing effect was significant (P<0.01) with respect to TDOM (mg/200 mg DM) and MBP (mg/200 mg DM) and was not evident only in case of lowest supplementation level (L_10_) tested in the present study.

The *in vitro* depression observed could be attributed to high fat and ADF content of the walnut cake. Goes *et al*.; Marcondes *et al*.[18,19] reported that OM digestibility is negatively correlated to high fiber content. It has been reported that walnut oil composition is dominated largely by unsaturated fatty acids (mainly linoleic together with lesser amounts of oleic and linolenic acids) along with minor proportions of tocopherols, phospholipids, sphingolipids, sterols, hydrocarbons and volatile compounds [20]. Further, it has been shown that high concentrations of free fatty acids in the rumen can alter digestion and appetite. It is well-known that high dietary levels of fats inhibit ruminal fermentation and thus diminish the utilization of dietary fiber [21-23]. It is also reported that [24] the fermentation pattern is affected by oil supplementation for concentrate-based diets.

Based on the results of *in vitro* trials, L_10_ ration with 10% inclusion level of walnut cake in composite ration (4% level in composite ration) was the obvious and only available choice for the level to be tested through feeding trial.

## Conclusion

It can be concluded that walnut cake incorporation up to 10% level in the iso-nitrogenous concentrate mixture has no any negative effect on *in vitro* digestibility of DM, TDOM, MBP, EMP and total gas production in goat.

## Authors’ Contribution

RKS conceptualized the aim of this study, designed and supervised the experiment. MAM executed the experiments, carried out laboratory analysis of feed samples and conducted statistical analysis. AR and KB has drafted and revised the manuscript. All authors read and approved the final manuscript.
